# EGR1 Transcription Factor is a Multifaceted Regulator of Matrix Production in Tendons and Other Connective Tissues

**DOI:** 10.3390/ijms21051664

**Published:** 2020-02-28

**Authors:** Emmanuelle Havis, Delphine Duprez

**Affiliations:** Laboratoire de Biologie du Développement, UMR7622, Institut Biologie Paris Seine, Sorbonne Université, CNRS, Inserm U1156, F75005 Paris, France

**Keywords:** connective tissues, tendon, adipose tissue, extracellular matrix, collagen, EGR1

## Abstract

Although the transcription factor EGR1 is known as NGF1-A, TIS8, Krox24, zif/268, and ZENK, it still has many fewer names than biological functions. A broad range of signals induce *Egr1* gene expression via numerous regulatory elements identified in the *Egr1* promoter. EGR1 is also the target of multiple post-translational modifications, which modulate EGR1 transcriptional activity. Despite the myriad regulators of *Egr1* transcription and translation, and the numerous biological functions identified for EGR1, the literature reveals a recurring theme of EGR1 transcriptional activity in connective tissues, regulating genes related to the extracellular matrix. *Egr1* is expressed in different connective tissues, such as tendon (a dense connective tissue), cartilage and bone (supportive connective tissues), and adipose tissue (a loose connective tissue). *Egr1* is involved in the development, homeostasis, and healing processes of these tissues, mainly via the regulation of extracellular matrix. In addition, *Egr1* is often involved in the abnormal production of extracellular matrix in fibrotic conditions, and *Egr1* deletion is seen as a target for therapeutic strategies to fight fibrotic conditions. This generic EGR1 function in matrix regulation has little-explored implications but is potentially important for tendon repair.

## 1. Tendon is a Proper Dense Regular Connective Tissue

Connective tissues support and link organs, and are composed of specialized fibroblasts derived from mesenchymal stem cells. A feature of connective tissues is the presence of extracellular matrix conferring specific biomechanical properties and functions, reviewed in [1]. Connective tissues include proper and supportive connective tissues, such as cartilage and bone. Proper connective tissues are further divided into two types: dense and soft/loose, reviewed in [2]. The dense connective tissues can also be divided into two subtypes: (1) the regular connective tissue, which refers to tendons/ligaments built with regular collagen fibers and (2) the irregular connective tissues embedding organs, composed of irregular collagen fibers, such as skeletal muscle connective tissue, pericardium, or peritoneum. Adipose tissue is a proper loose connective tissue mainly composed of adipocytes, included in a disorganized network of collagen fibers. Connective tissues contain fibroblasts described as interstitial cells producing extracellular matrix. However, fibroblasts display remarkable heterogeneity of molecular signatures and phenotypes across connective tissues, reviewed in [3] and fibroblast populations are still not well characterized. Fibroblast deregulation leads to fibrosis, assessed by excessive deposition and anarchic organization of extracellular matrix (ECM).

Tendon is a proper dense regular connective tissue that links muscle to bone and is involved in the transmission of forces generated by muscle contraction to bone. Tendon is a key component of the musculo-skeletal system, which allows body movement. Although tendon stem cells have been identified [4], the molecular identity of tendon cells is not well defined and the understanding of tendon biology lags behind that of other organs. Researchers in the tendon field eagerly await the analysis of tendon transcriptomic single-cell data to characterize tendon cell propulations. The main structural and functional component of the tendon is type I collagen. However, the presence of type I collagen is not specific to tendon since it is expressed in many other connective tissues such as bone and adipose tissue. Tendon specificity is given by the spatial organization of type I collagen fibrils paralel to the tendon axis. A multitude of matrix molecules are involved in collagen fibrillogenesis leading to the specific spatial organization of type I collagen in tendons, reviewed in [5,6]. To date, the bHLH transcription factor Scleraxis (SCX) and the transmembrane protein tenomodulin (TNMD) are recognized as matrix regulators in tendons during development, homeostasis, and repair, as reviewed in [7] for SCX and in [8] for TNMD. SCX is recognized to regulate *Tnmd* expression [9,10]. However, these two genes are not fully specific to tendons, since they are expressed in other connective tissues. *Scx* is also expressed in heart valves [11], muscle connective tissue [12], and fibroblasts of kidney, testis, and lung [13]. In addition to being expressed in tendon, *Tnmd* is also expressed in dermis, brain, and adipose tissue, reviewed in [8]. In addition to SCX, two other transcription factors have been shown to positively regulate the expression of *Col1a* and *Col1a2* genes and type I fibril organization in tendons: the homeobox Mohawk (MKX) and the zinc finger transcription factor Early growth response gene 1 (EGR1). However, *Mkx* and *Egr1* are also not specific to tendon, since they display numerous expression sites and have been shown to be involved in mutiple biological processes, reviewed in [14,15].

**Figure 1:**
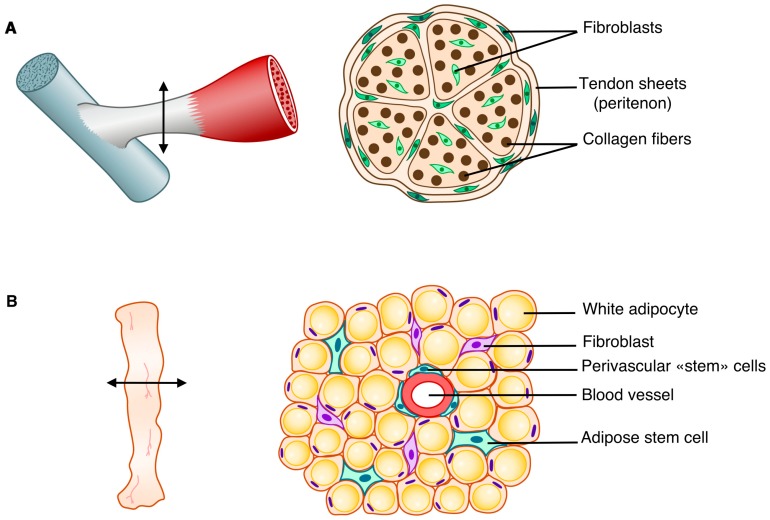
Schematic representation of two connective tissues: a dense regular connective tissue, the tendon (**A**), and a loose connective tissue, the adipose tissue (**B**). Left panels represent tendon (**A**) and adipose tissue (**B**). Arrows in left panels indicate the section levels in each tissue. Right panels show the cellular composition on sections of tendon (**A**) and adipose tissue (**B**).

In this review, we will focus on the EGR1 transcription factor and its generic function as a regulator of gene transcription of extracellular matrix components in tendon, bone, and adipose tissue (Figure 1), both in physiological and pathological conditions. 

## 2. *Egr1* “Identity Card”

### 2.1. Multiple Names for a Single Gene

The *Egr1* (*Early growth response 1*) gene was first identified as *NGFI-A* (*Nerve Growth Factor Induced-A*) because of its activation by NGF (nerve growth factor) in the neuronal rat cell line PC12 [16]. It was subsequently described in four different laboratories as a rapidly and transiently activated gene in various fibroblast cell lines. Serum addition or mitogen treatment with the tumor promoter TPA (tetradecanoyl phorbol acetate) on mouse fibroblasts led to a rapid and strong induction of an early growth response gene then named *Egr1* [17,18]. *Egr1* was also named *TIS8* (*TPA Inducible Sequence 8*), because of its activation following TPA treatment in a murine 3T3 cell line [19]. After the identification of a zinc-finger-region similar to the Drosophila Krüppel segmentation gene in the *Egr1* genomic sequence, the murine gene was called *Krox24*, for “Krüppel box 24” [20] or *zif/268* in reference to three tandem zinc finger sequences [21]. Presentation of recorded bird songs to songbirds such as canaries and zebra finches induces *Egr1* expression in their forebrains. In this case, *Egr1* was referred to by the acronym *ZENK* [22]. The *Egr1/EGR1* gene spans about 3.8 Kb and is located on chromosome 18 in mice and on chromosome 5 in humans. It is composed of two exons and one 700 bp intron. The first exon includes the first 99 amino acids of the protein and the second exon includes the three tandem zinc finger motifs [23]. The *Egr1/EGR1* gene is highly conserved between mouse, rat, chicken, zebrafish, chimpanzee, dog, cow, and human. 

### 2.2. Numerous Extracellular Signals Regulate Egr1 Expression via Diverse Intracellular Signalling Pathways

**Figure 2:**
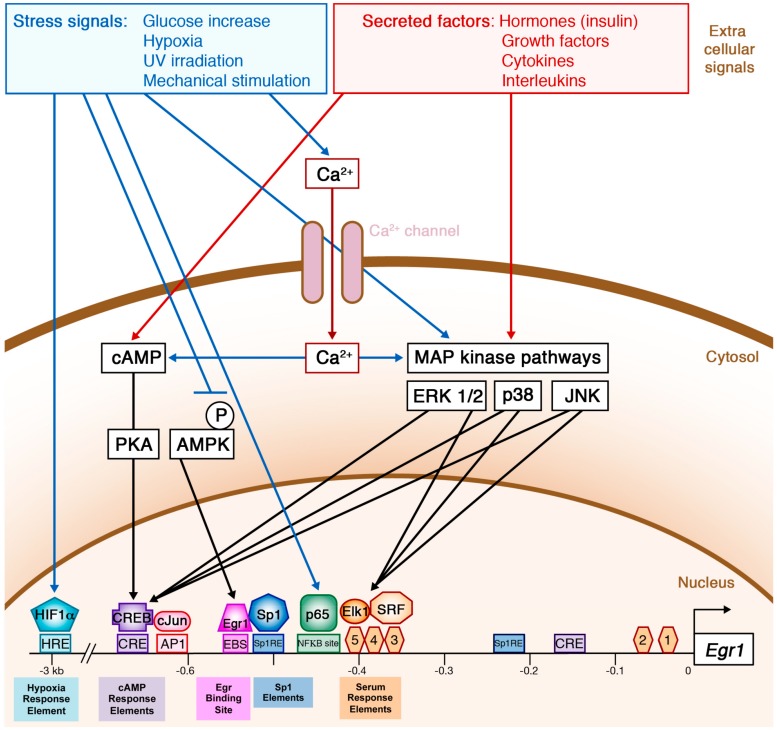
Schematic representation of the external stimuli that regulate *Egr1* transcription.

The analyses of 5′-upstream sequences of murine *Egr1* and human *EGR1* genes reveal the conserved presence of many regulatory elements, indicating that *Egr1* expression is regulated in response to multiple stimuli. The seemingly endless list of extracellular stimuli inducing *Egr1* transcription include hormones, growth factors, cytokines, UV, hypoxia, stress, and mechanical signals (Figure 2). The MAPK (mitogen-activated protein kinase) signalling pathways has a central role downstream of these external stimuli to regulate *Egr1* expression. *Egr1* expression is activated by a variety of MAPK-inducing factors, including TNFs (tumor necrosis factors) in primary human fibroblasts and rat chondrocytes [24,25], different pro-inflammatory cytokines IL-1, TNFα, and IL-17 in osteoblast-like cells [26], FGF21 in mouse adipose tissue [27], and the antimicrobial peptide LL-37 in adipose stem cells [28]. These secreted molecules activate the MAPK pathways, which leads to the recruitment of the SRF (serum response factor) transcription factor together with the ternary complex factor (TCF)/Ets family member ELK1 to the serum response elements (SRE) of *Egr1* promoter [29]. The contribution of individual SRE in *Egr1* transcriptional activation depends on cell types [30,31]. Interestingly, TGFβ upregulates *Egr1* expression through a Smad-independent pathway in human fibroblasts, and activates the MEK1/2/ERK signalling cascade converging on ELK-1 to induce *Egr1* expression [32]. The MKK7-JNK-cJun MAPK pathway can also activate mouse *Egr1* expression through the formation of the AP1 complex [33]. AP1 complexes are heterodimeric transcription factors that bind to Tetracecanoyl Phorbol Acetate Response Elements, also called AP1 elements. AP1 complexes are composed of combination of proteins belonging to the c-Fos, c-Jun, activating transcription factor (ATF), and Jun dimerization protein families reviewed in [34]. cJun directly binds to the AP1 element of the *Egr1* promoter but not to the SREs, meaning that cJun directly activates *Egr1* expression via the AP1 site and indirectly via the SRE sites [33]. 

Analysis of deletions in the human *EGR1* and mouse *Egr1* promoters in fibroblasts revealed that AP1 and EGR1 binding sites played minor roles in its activation [35]. EGR1 binds to its own promoter in mouse fibroblasts [36]. This finding leads to the observation that the ubiquitously expressed transcription factor SP1 (specificity protein 1) binds to a consensus recognition sequence that partially overlaps with the EGR1 binding site (EBS) [36] and blocks EGR1 recruitment to EBS (Figure 2). Serum stimulation leads to *Egr1* overexpression, which binds competitively with SP1 to EBS. This binding of EGR1 to its own promoter downregulates *Egr1* expression [37], presumably through the MAPK pathways. In addition to MEK/ERK pathways, the cAMP/PKA/CREB pathway mediates *Egr1* activation downstream of hormones or cytokines [38,39,40]. 

*Egr1* expression is rapidly but transiently induced by insulin in 3T3-L1 adipose cell line and in mouse adipose tissue [41]. Insulin secretion induces a cascade of intracellular events in the adipocyte cell lineage [42] including the PI3K/Akt pathway responsible for glucose uptake and glycogenesis and the ERK1/2 MAPK pathway, which decreases insulin signalling [43,44]. Glucose stimulation induces a rise in cytoplasmic Ca^2+^ which is necessary for AP1, CRE, and SRE-mediated activation of *Egr1* expression [45]. A high insulin and glucose concentration represses the phosphorylation of AMP-activated protein kinase (AMPK) in rat tenocytes, which prevents its positive role in *Egr1* expression and alters tendon homeostasis [46]. 

Several stress signals, such as UV, hypoxia, and mechanical shear stress have been shown to activate *Egr1/EGR1* transcription. UV irradiation activates *Egr1* expression in multiple cell types, including mouse and human fibroblasts as well as human fibrosarcoma cells [47]. The UV-B effect on *Egr1* expression is mediated through the activation and recruitment of the NFκB family member p65 to a NFκB binding site located on the *Egr1* promoter [48]. Under hypoxic conditions, the hypoxia-inducible factor-1α (HIF1α) binds to the *EGR1* promoter in adipose stem cells isolated from obese patients, but not not in hASCs from healthy patients [49]. Hypoxia induces *EGR1* upregulation in hASCs of diabetic patients either through the MAPK/ERK pathway or via the direct recruitment of HIF1α to the *EGR1* promoter [49]. Fluid shear stress activation activates *EGR1* transcription in human endothelial cells and epithelial cells [50]. 

In summary, *Egr1* expression is regulated by a myriad of secreted molecules and stress factors through numerous regulatory elements located upstream of the *Egr1* coding sequence. 

### 2.3. EGR1 Protein: Structure and Transcriptional Activity 

**Figure 3:**
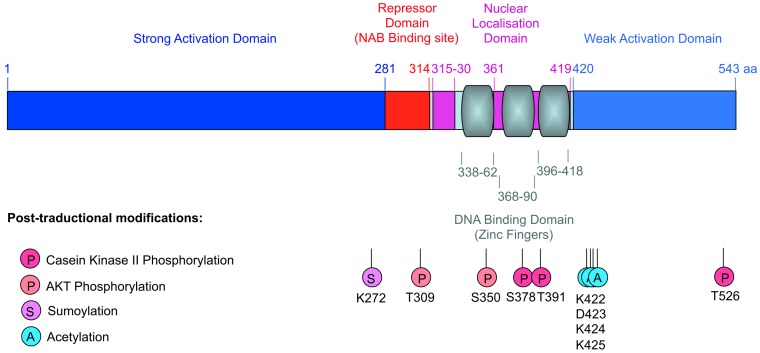
Schematic representation of the post-translational modifications on EGR1 protein.

The EGR1 protein contains 533 and 543 amino acids in mice (Figure 3) and humans, respectively, with a predicted molecular weight of 58 kDa. However, EGR1 detection by Western-blot analysis shows an apparent molecular weight running from 80 to 100 kDa, presumably due to post-translational modifications [24,36,51]. EGR1 contains a highly conserved DNA-binding domain composed of three Cys2-His2 type zinc fingers [52]. These three zinc fingers recognize a consensus nine-base-pair segment of a G/C rich region of DNA (5′-GCG(C/G/T)GGGCG-3′), with each zinc finger spanning three nucleotides [36,52,53]. EGR1 also contains a bipartite nuclear localization domain, a strong activation domain, a weak activation domain, and an inhibitory domain [54] on which NAB1 and NAB2 (NGF1-A binding proteins) bind to repress transactivation by EGR1 [55,56]. According to EGR1 structure, EGR1 displays activator or repressor transcriptional activities [55,56,57]. 

The phosphorylation of the different EGR1 domains (Figure 3) is controlled by protein kinases and phosphatases [58]. Phosphorylation can either enhance or block EGR1 transcriptional activity. In fibrosarcoma, UV exposure leads to EGR1 phosphorylation by PKC (protein kinase C) and tyrosine kinases [59], conferring on EGR1 a protective and anti-apoptotic function [60]. In contrast, EGR1 phosphorylation by the protein kinase CKII has a negative effect on EGR1 DNA binding and transcriptional activities [61]. EGR1 can be acetylated (Figure 3) by the CBP/p300 complex [62]. In summary, EGR1 activates or represses specific genetic programs according to its “phosphorylation/acetylation pattern”. 

EGR1 can be multi-ubiquitinated and thus degraded by the ubiquitin-dependant proteasome pathway [63]. The coordinated sumoylation (Figure 3) and ubiquitination of EGR1 by SUMO-1 (Small Ubiquitin like MOdifier 1) and UBC9 (ubiquitin conjugating enzyme 9) have been shown to be involved in EGF-induced *EGR1* expression and stability in the human endothelial cell line ECV304 [64]. Redox (Reduction-Oxidation) reactions are involved in various vital cellular functions, such as aerobic cellular respiration, nucleic acid synthesis, and also for the production and elimination of reactive oxygen species (ROS), which includes superoxide, nitric oxide, hydroxyl radical, hydrogen peroxide, and hypochlorus acid. At high doses, ROS are toxic for the cell and the damage they cause was termed oxidative stress. Oxidative stress induces bone loss by stimulating osteoclastic bone resorption and inhibiting osteoblastic differentiation [65,66,67,68]. However, hydrogen peroxide at non-toxic doses increases the expression and DNA-binding activity of EGR1 in mouse and human osteoblastic cells without affecting their differentiation [69,70]. The DNA-binding properties of EGR1 are modulated by the redox state: EGR1 binding to the DNA depends on the presence of reducing agents, which are necessary for the correct conformation of the EGR1 zinc-finger region. Oxidized or metal-free EGR1 does not bind to DNA [71]. In the human osteoblastic HOBIT cell line, under non-toxic ROS doses, a DNA repair enzyme, the APE1 (apurinic/apyrimidic endonuclease 1) increases EGR1 binding to DNA with nuclear redox activity. EGR1 also upregulates *APE1* gene expression, showing the existence of a positive-autoregulatory loop between APE1 and EGR1 proteins [70]. 

In summary, EGR1 displays diverse transcriptional activation or repression functions depending on its post-translational modification statues. 

### 2.4. Egr1 Expression Profile In Vivo 

*Egr1* is expressed in numerous organs and cell types during development and adult life. However, *Egr1* expression is not ubiquitous. In situ hybridization experiments performed on chicken and mouse embryos indicate a punctiform location of *Egr1* transcripts in various tissues such as tendon, cartilage, bone, skeletal muscle, innervation, vessels, and dermis [72,73]. In developing tendons, *Egr1* transcripts are not observed in all tendon cells, but in subregions, such as the myotendinous junction and around long tendons in mouse and chicken embryos [73,74]. In the adult, the mouse ENCODE transcriptome data set indicates that *Egr1* is expressed in many if not all adult tissues, with high expression in cortex, mammary gland, ovary, and thymus [75]. In situ hybridization and immunohistochemistry experiments performed on adult mouse tissues show *Egr1* expression in Achilles tendons [76], subcutaneous adipose tissue [77], hypertrophic cartilage [78,79], and bone [80,81].

## 3. EGR1 Roles in Connective Tissue Formation, Homeostasis and Healing

Consistent with the broad range of *Egr1/EGR1* expression sites, EGR1 is involved in the formation and homeostasis of many organs. One powerful tool for addressing gene function is the use of knock-out mice. The *Egr1* gene was inactivated in mice by homologous recombination with the insertion of the neomycin resistance cassette upstream of the EGR1 DNA-binding domain [82] and with the insertion of the *LacZ* coding sequence within the *Egr1* 5′ untranslated region added with a frameshift mutation upstream of the DNA-binding domain of *Egr1* [83]. Both *Egr1* mutant mouse lines were initially described with no overt phenotype during development or postnatal life [82,83]; with the exception of subtil pituitary and ovarian defects observed in the *LacZ* insertion mutant mice [83]. However, connective tissue defects were subsequently described in these *Egr1* mutant mice, affecting tendon, cartilage, bone and adipose tissue formation and homeostasis. The analysis of *Egr1* loss-of-function in mice has identified numerous EGR1 target genes in connective tissues (reported in Table 1). A striking point is that the target genes positively regulated by EGR1 are mainly components of the extracellular matrix (ECM) or linked to ECM regulation, while those negatively regulated by EGR1 are cartilage, bone, or adipose tissue differentiation markers (Table 1). 

### 3.1. EGR1 Is a Potent Inducer of Extracellular Matrix Production in Tendons 

#### 3.1.1. EGR1 Function in Tendon Formation, Homeostasis, and Ageing 

Several studies have addressed the role of EGR1 in tendon biology. In developing limb tendons, *Egr1* is expressed close to the myotendinous junction and delineates the long tendons in mouse and chicken embryos [73,74]. The *Egr1* mutant mice do not display a strong overt tendon phenotype; however, *Egr1^−/−^* mice show a significant decrease in the expression of the key tendon markers, *Scx, Tnmd,* and *Col1a1* in addition to that of tendon-associated collagens in developing E18.5 limbs [73] and adult tail tendons and Achilles tendons [76]. Comparison of adult tail tendons in *Egr1^−/−^* versus *Egr1^+/+^* mice shows a reduced number of collagen fibers in mutant mice. Tail and Achilles tendons have collagen fibrils with smaller diameter and impaired biomechanical properties in *Egr1^−/−^* compared to *Egr1^+/+^* mice [76]. Conversely, *Egr1* is sufficient to induce de novo expression of a large variety of tendon genes (including *Scx* and tendon-associated collagen genes) in ectopic contexts in chicken embryos [73]. The *Egr1* gene is sufficient to induce ectopic *Scx, Col1a1, Col3a1*, *Col5a1,* and *Col14a1* expression in the neural tube, an unrelated embryonic tissue derived form the ectoderm [73]. Consistently with the in vivo situation, EGR1 is sufficient to induce the expression of a large panel of tendon genes including, *Scx* and *Tnmd*, collagen associated-tendon genes (*Col1a1, Col1a2 Col3a1, Col5a1, Col6a1, Col14a1*), and tendon matrix-associated molecules (*Tnc, Bgn, Dcn*, and *Fbn1*) in mouse C3H10T1/2 mesenchymal stem cells [76]. EGR1 also promotes the formation of 3D-engineered tendon constructs made of C3H10T1/2 cells by increasing the expression of *Scx*, *Tnmd*, and *Col1a* genes [76,85]. Moreover, EGR1 induces tenogenic differentiation in rabbit tendon stem cells [97]. EGR1 mediates the promoting effect of the anti-miR124 on collagen production in human tendon-derived stem cells [98] and the promoting effect of ferulic acid on self-renewal ability of human tendon-derived stem cells [99]. Consistent with the positive regulation of *Col1a* gene transcription by EGR1 observed both in vivo and in vitro, chromatin immunoprecipitation (ChIP) experiments show the recruitment of EGR1 to the tendon regulatory regions of the *Col1a1* promoter in E18.5 limbs [73] and to *Col1a1* and *Col1a2* regulatory regions in adult Achilles tendons [76]. Lastly, *Egr1* downregulation has been associated with a loss of the tenogenic differentiation potential in ageing human tendon progenitor cells, while *Egr1* gain-of-function has the ability to rescue their tendon differentiation potential as assessed by the upregulation of *Scx, Tnmd, Bgn, Dcn,* and *Col1a1* gene expression [84]. 

#### 3.1.2. EGR1 Function in Tendon Healing

EGR1 has been shown to be required for the expression of the key tendon markers and tendon-related ECM genes during healing after tendon injury [76]. Tendon injury models where the tension is maintained (partial rupture) induce a massive increase of tendon gene expression [76,100], while tendon injury models where the tension is lost (total rupture) induce a loss of tendon gene expression [101,102]. Different models of tendon injury, where tension is maintained, lead to a massive increase of *Egr1* expression after injury, in mouse or rat Achilles tendons [76,100] and rabbit flexor tendons [103]. In addition, needle-induced microlesions in healing rat Achilles tendons increase *Egr1* expression [104]. The transcriptional response i.e., the increase of *Scx, Tnmd, Col1a1, Col1a2, Col5a1, Col6a1, Col14a1*, *Tnc,* and *Dcn* gene expression in response to longitudinal lesion along the tendon axis is drastically decreased in *Egr1^−/−^* Achilles tendons, showing that *Egr1* is required for the injury-induced expression of tendon-related ECM genes and key tendon markers [76]. Moreover, EGR1-producing cells promote tendon repair in a rat model of Achilles tendon injury [76] and in a rabbit model of rotator cuff injury [97]. 

In summary, *Egr1* appears to be required for the correct expression of matrix genes during tendon formation, homeostasis, and ageing in vitro and in vivo, but also during tendon healing. A recent discovery is that tendons are peripheral circadian clock tissues, reviewed in [105], in which collagen synthesis and homeostasis is under a circadian control [106]. Interestingly, the circadian clock is disturbed in *Egr1^−/−^* mice, associated with impaired locomotor activity and body temperature [107], suggesting that *Egr1* could be involved in the circadian synthesis of tendon-associated collagens.

### 3.2. Egr1 Is a Mechanosensitive Gene in Tendon

Due to its function to transmit load from muscle contraction to the skeleton, tendon is a mecanosensitive tissue. Mechanical signals are necessary parameters involved in tendon development, homeostasis, and healing, reviewed in [108,109,110]. Mechanical tendon properties have been identified in developing embryonic tendons [111]. Muscle contractions are required for tendon development. In the absence of muscles or muscle contractions, head, axial, and limb tendons do not form; this muscle-dependency for tendon formation is observed in zebrafish, chicken, and mouse embryos, reviewed in [14]. TGFβ and FGF signalling pathways prevent tendon gene downregulation in paralyzed developping limbs in chicken embryos [112]. Since EGR1 has been shown to directly activate *Tgfb2* transcription in mouse developping limbs [73], EGR1 is one possible mechanosensor protein downstream of mechanical forces and upstream of TGFβ signalling in developing limbs. Consistent with this mechanosensor function, *Egr1* is activated by mechanical loading in 3D-engineered tendons made of human tendon cells [113] and equine tenocytes derived from induced-pluripotent stem cells [114]. Moreover, EGR1 regulates tendon gene expression downstream of mechanical signals in 3D-engineered tendons made of mouse mesenchymal cells [85,113]. Forced-EGR1 expression prevents the downregulation of the tendon genes, *Scx, Tnmd, Col1a1, Col1a2,* and *Tgfb2* in 3D-engineered tendons after tension release [85]. Mechanical signals are also required for tendon homeostasis. Loss of mechanical loading induces tendon defects is reviewed in [108,115]. Botox (botuliniumtoxin A) injection into the gastrocnemius muscle of hindlimb induces a decrease of *Egr1* transcription in addition to *Scx, Col1a2,* and *Tgfb2* transcription in mouse Achilles tendons [85]. It is also recognized that the lack of mechanical stimulation is deleterious for tendon healing, while mechanical stimulation improves tendon healing in rats, mice, and humans [108,115]. *Egr1* expression is increased within 15 min in response to loading during the healing process in injured rat tendons [100], indicating that *Egr1* expression reflects a rapid transcriptional response following loading changes in healing tendons. The increase of *Egr1* expression in injured Achilles tendons after mechanical loading is followed by an increase of tendon strength, indicating a beneficial role for *Egr1* in tendon healing [100]. These observations confirm the mechanosensor role for EGR1 during tendon healing. Conversely, *Egr1* expression is decreased in a mouse Achilles tendon injury model in reduced load conditions [85]. The *Egr1* downregulation is concomitent with the downregulation of tendon genes *Scx*, *Tnmd*, *Col1a1*, *Col1a2,* but also that of *Tgfb2* genes [85]. EGR1 rescue experiments in reduced load conditions and after tendon injury increase the expression of tendon-associated genes, including *Scx*, *Tnmd*, *Col1a1*, *Col1a2,* and *Tgfb2* [85]. These observations suggest that *Egr1* is sufficient to drive the tendon differentiation program in the absence of mechanical signals during the healing process in vivo.

**Figure 4:**
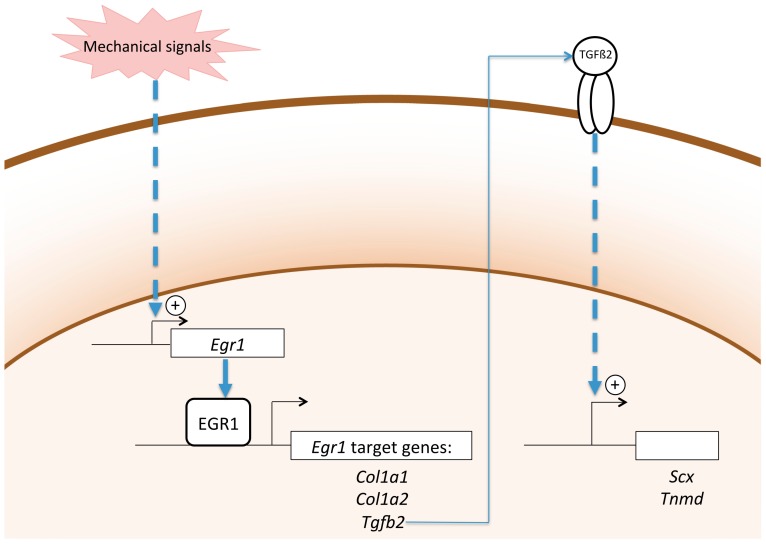
Schematic representation of the position of *Egr1*/EGR1 in the molecular cascade downstream of mechanical signals involved in tendon gene expression.

Mechanical signals are the driving force for tendon cell differentiation in different contexts. Although the scheme is not complete, one attempt to hierarchize mechanical and molecular signals would be that mechanical signals activate molecular signals such as EGR1 transcription factor, which then in turn activate the expression of tendon genes such as *Scx* and *Tnmd* and *Col1a* genes (Figure 4). It has to be noticed that *Tgfb2* transcription is also induced by EGR1 rescue experiments in unload conditions in a mouse model of Achilles tendon injury and 3D-engineered tendons [85]. This data, combined with the direct binding of EGR1 to *Tgfb2* promoter regions in adult tendons [76], leads to the hypothesis that EGR1 activates *Tgfb2* dowstream of mechanical signals, which in turn will activate tendon matrix genes (Figure 4). This is consistent with the recognized role of TGFβ signalling in the control of tendon adaptation to mechanical loading, reviewed in [116]. The link between EGR1 transcription factor and the YAP key mechanotransduction pathway [110] remains to be established.

### 3.3. EGR1 and Endochondral Bone Formation and Healing

Bone is a supportive connective tissue composed of cells, fibers, and a mineralized solid ground substance [117]. *Egr1* expression is detected in several areas undergoing endochondral bone formation, such as hypertrophic cartilage [78,79] and periostal regions of the developping long bones [72,73]. *Egr1* is a negative regulator of cartilage markers [92]. *Egr1^−/−^* mice display bone loss [80,118,119]. The bone loss upon *Egr1* deletion is a consequence of an increased bone resorption, via the increased production of the colony stimulating factor-1 CSF-1/M-CSF known to positively regulate osteoclast differentiation [80]. Bone development and homeostasis is controlled by the interplay between bone-forming osteoblasts and bone-resorbing osteoclasts [120], and this equilibrium is lost in *Egr1^−/−^* mice. EGR1 is a negative regulator of the osteoclastogenic cytokine CSF-1 production by stromal cells. Phosphorylated-EGR1 (upon estrogen) blocks *Csf1* gene transcription by preventing the binding of the transcriptional activator SP1 to the *Csf1* promoter [80]. The failure of estrogen to rescue the CSF-1 production and consequent osteoclast formation in *Egr1^−/−^* in ovariectomized mice positiones *Egr1* as a pivotal actor to mediate the anti-osteoclastogenic effect of estrogen [80,81]. 

Transcription factors that play a role in bone formation are expected to participate in bone healing after fracture since endochondral bone formation that occurs in embryos is recapitulated during bone healing. In a bone fracture mouse model, *Egr1* deficiency leads to several bone defects including persistant fibrin accumulation in the fracture gap, abnormal callus ossification with enlarged areas of cartilaginous tissue, decreased expression levels of *Bglap* (Osteocalcin), and bone resorbtion markers that regulate extracellular matrix, including *Acp5* (Tartrate-resistant acid phosphatase) and *Ctsk* (Cathepsin K) genes [93,118]. This data confirms that EGR1 controls the balance between bone tissue formation and resorption during skeletal repair.

### 3.4. EGR1 Regulates Extracellular Matrix Production in Adipose Tissue 

Adipose tissue is a loose connective tissue mainly composed of specialized white adipocytes, held in a framework of collagen fibers, which plays a fundamental role in fat storage, metabolic control, and thermoregulation [121]. Adipocytes are surrounded by an extracellular matrix, which serves as mechanical support and is mainly composed of collagens, fibronectin, and elastin [122]. Cells producing ECM in adipose tissue are not clearly indentified; however, it is recognized that collagens are mostly produced by adipocytes, but also by endothelial cells and adipose stem cells in normal conditions [123]. The molecular signature of subcutaneous white adipose tissue with *Egr1* deletion identifies a downregulation of ECM genes, including *Col1a1*, *Col1a2, Col3a1, Col5a1, Col5a2*, *Col14a1*, *Fn1*, *Dcn,* and *Post* (*Periostin*) [77]. *Egr1* deletion is also associated with a spontaneous browning of subcutaneous white adipose tissue in *Egr1* mutant mice compared to wild-types [77]. The browning phenotype corresponds to the appearence of beige adipocytes within the white adipose tissue mainly by de novo differentiation of progenitors [124]. In contrast to white adipocytes, beige adipocytes dissipate excess energy through heat production by a large number of mitochondria, which exhibit uncoupling activity via the thermogenic protein UCP1 (uncoupling protein-1) [125]. Consistent with the browning phenotype, transcriptomic analysis in *Egr1*-deleted adipose tissue shows a concomitant downregulation of the white adipocyte marker, *Lep* (leptin) and upregulation of the key beige adipocyte marker *Ucp1* [77]. Both positive (*Lep*) and negative (*Ucp1*) transcriptional regulations have been shown to occur via direct EGR1 recruitment to regulatory regions of these genes [41,77]. The browning process reduces the deleterious consequences of fat accumulation and is seen as possible mechanism to fight against obesity and to improve metabolic health [125]. Consistently, *Egr1^−/−^* mice are protected from high fat diet-induced obesity via an increase of energy expenditure [95]. Conversely to *Egr1* loss-of-function in mice, *Egr1* overexpression in C3H10T1/2 cells increases the transcription of matrix genes and prevents the beige adipocyte differentiation [77]. Overall, *Egr1* deletion leads to a drastic loss of ECM genes associated with a browning phenotype in subcutaneous white adipose tissue in mice. 

To summarize, *Egr1* loss-of-function is associated with reduced ECM production in tendon (a dense connective tissue), bone (a supportive connective tissue), and adipose tissue (a loose connective tissue). Although the altered matrix genes upon *Egr1* deletion appear to be similar in the different connective tissues, the reduced ECM production is deleterious for tendon and bone formation and homeostasis, while being beneficial for white adipose tissue to increase energy expenditure. 

## 4. EGR1 Is a Fibrotic Factor 

Fibroblast deregulation leads to fibrosis, a process attributed to anarchic deposition of extracellular matrix, in response to injury or in pathological conditions. Fibrosis is observed in almost any tissue in cases of organ dysfunction, but is also a key process in cancer, inflammation, and ageing. Myofibroblasts are the main cellular component of fibrosis. Myofibroblasts are non-muscle contractile cells responsible for the excessive synthesis, deposition, and remodelling of ECM proteins in fibrosis; for a recent review see [126]. A recognized molecular driver of fibrosis is the TGFβ signalling pathway. TGFβ drives the conversion of fibroblasts to myofibroblasts to induce the excessive deposition of collagen and inappropriate ECM during fibrosis, recently reviewed in [127]. Generic molecular markers for myofibroblasts are similar to markers for smooth muscle cells, such as *Acta2* (smooth muscle actin), whose expression is regulated by TGFβ signalling [127]. Although myofibroblasts and TGFβ are recognized to be the respective cellular and molecular hallmarks of fibrosis, there is no comprehensive understanding of the cellular and molecular mechanisms underlying fibrosis. As EGR1 is a potent regulator of matrix components in different contexts, EGR1 is seen as a fibrotic factor. 

### 4.1. EGR1 and Fibrosis in Metabolic Diseases Linked to Adipose Tissue (Obesity and Diabetes)

Fibrosis in adipose tissue is considered a hallmark of metabolically dysfunctional adipose tissue and is associated with obesity and insulin resistance [86,122,128]. In pathological conditions such as obesity, the rapid expansion of adipose tissue causes hypoxia since neovascularization cannot keep up with rapid adipose tissue growth. Hypoxia is followed by the necrosis of adipocytes accompanied with the infiltration of inflammatory leucocytes and macrophages to remove the dead cells. In these pathological conditions, myofibroblasts associated with the inflammatory response, accumulate within the adipose tissue and cause ECM thickening, characteristic of fibrosis [121,129]. Adipose tissue fibrosis results from the imbalance between excess synthesis and impaired degradation of type I, III, and VI collagens [130]. Consistently, type I, III, and VI collagens are particularly abundant in adipose tissue of obese patients [131] and collagen content affects tensile strengh of adipose tissue [132]. Moreover, *Col6a1* loss-of-function significantly reduces adipose tissue fibrosis in obese mice [122]. *Egr1/EGR1* expression is increased in adipose tissue of obese *Lep/Lep* mice and patients [95], and of diabetic *db/db* mice and type 2 diabetes mellitus (T2DM) patients [133]. T2DM is associated with insulin resistance and the majority of T2DM patients are overweight or obese [129]. *Egr1* gain-of-function in epididymal fat induces insulin resistance [133], while *Egr1* loss-of-function improves the whole-body insulin sensitivity in diabetic mice [133]. One possible mechanism would be that insulin-induced EGR1 [41,133] directly inhibits *Pnpla2* (*ATGL, Adipose TriGlyceride Lipase*) expression in adipocytes that leads to lipolysis inhibition and promote fat accumulation [94].

*Egr1* overexpression is associated with metabolically dysfunctional adipose tissues. As *Egr1* regulates matrix production in subcutaneous white adipose tissue and tendon, *Egr1* is a credible regulator of inappropriate ECM production in metabolically dysfunctional adipose tissues. Consistent with this idea, *Egr1* transcription is directly activated by insulin and HIF1α [49] via direct recruitment to *Egr1* promoter. In addition, the insulin and HIF1α expression is increased in obese and T2DM mice and HIF1α regulates the expression of ECM genes such as *Col1a1, Col3a1,* and *Col14a1* [86,87]. *Egr1* is thus a good therapeutic target to counteract obesity and associated fibrosis since its loss-of-function reduces ECM production and stimulates the white fat browning process.

Interestingly, the tendon differentiation gene, *Tmnd*, involved in ECM regulation in tendons [8], is also involved in adipose tissue function. *TNMD* mRNA expression levels are strongly correlated with the body mass index. *TNMD* gene expression is significantly higher in obese subjects compared to lean subjects [134,135,136], while *TNMD* gene expression is downregulated in visceral adipose tissue during diet-induced weight loss [134]. Although *EGR1* and *TNMD* genes share the same expression profile in white adipose tissues of obese patients, in contrast to *EGR1*, *TNMD* acts as a protective factor in visceral adipose tissue to alleviate insulin resistance in obesity [136]. 

### 4.2. Tendon Defects in Type 2 Diabetes Mellitus

Type II diabetes mellitus (T2DM) is associated with high risk of tendinopathy or tendon tears, reviewed recently in [137,138]. Various T2DM rodent models, which cannot be dissociated from obese mouse models, display tendons with decreased collagen content, ECM disorganization, and impaired mechanical properties, reviewed in [138]. T2DM also impairs the healing process following tendon injury and amplifies the fibrotic process during healing, leading to scarred tendons. There is an increase of *Col1a1* and *Col3a1* expression in FDL tendons after injury associated with decreased mechanical properties in diet-induced obesity mice [88]. High glucose also affects tendon gene expression and cell behavior in tendon cell cultures, which induces changes in extracellular matrix [139]. Interestingly, *Egr1* expression was also modified (associated with *Mkx*, *Tgfb1*, *Col1a2*, and *Bgn* expression alteration) in rat tendon cells cultured in high glucose for 14 days [46]. 

### 4.3. EGR1 Controls Fibrosis in Systemic Sclerosis

A classical fibrotic disease is systemic sclerosis, also known as scleroderma, which is a rare disease characterized by excessive collagen deposition resulting in fibrosis in different organs such as skin and lungs but also the diggestive track (esophagus, stomach, and intestine) and myocard [140,141]. Systemic sclerosis pathology also displays inflammation and vasculopathy components. Myofibroblasts are key cells of the physiopathology of systemic sclerosis [140]. TGFβ and Wnt signalling are recognized to play a fundamental role in the pathogenesis of fibrosis in systemic sclerosis, in particular in the differentiation process of activated myofibroblasts [141].

EGR1 transcription factor has been shown to be at the crossroad of the molecular processes leading to the TGFβ-dependent fibrosic process in systemic sclerosis, reviewed in [142,143]. *EGR1* expression is increased in biopsies of fibrotic skin and lung from patients with systemic sclerosis [32]. In the mouse model of bleomycin-induced scleroderma, fibrosis is reduced in skin and lung of *Egr1^−/−^* mice, with decreased *Col1a1* expression and αSMA+ fibroblasts in both tissues [89]. In human fibroblasts, EGR1 upregulates *COL1A2* transcription downstream of TGFβ [91]. The high throughput analysis of EGR1-responsive genes in human primary fibroblasts identifies over 600 genes involved in extracellular matrix synthesis, wound healing, and TGFbeta signalling, but also in cell proliferation and vascular development [144]. This EGR1-responsive genes signature is enriched in skin biopsies from patients with systemic sclerosis compared to healthy controls [144]. The demonstrated involvement of EGR1 in this fibrotic disease identifies EGR1 as a pertinent target to control fibrosis in systemic sclerosis [142,143]. 

### 4.4. EGR1 Is at the Crossroad of the Molecular Pathways Involved in the Fibrotic Process in Animal Models for Organ Fibrosis

Classical animal models for fibrosis target idiopathic pulmonary fibrosis, renal fibrosis, liver fibrosis, and heart fibrosis. EGR1 is frequently mentioned as being associated with the progression of fibrotic process in animal models and in the transcription of fibrotic genes in cellular models. *Egr1* deletion is often described as being beneficial to fight fibrosis progression in animal models of organ fibrosis.

*Lung fibrosis.* EGR1 is involved in lung fibrosis downstream of IGFBP-5 (insulin-like growth factor (IGF) binding protein-5) to promote fibrotic gene transcription [145] and downstream of TGFβ1 to activate the transcription of the hyaluronan receptor CD44V6 (CD44 containing variable exon 6 (v6)) expression in lung fibroblasts in the context of idiopathic pulmonary fibrosis [146].

*Renal fibrosis.* In a mouse model of adenine-enriched diet induced tubulointerstitial nephritis leading to renal fibrosis, *Egr1* is increased in kidney. *Egr1^−/−^* mice display reduced TGFbeta activity and reduced renal fibrotic zones and were protected from renal failure [147]. The miR181 was identified as an inhibitor of renal fibrosis via *Egr1* inhibition, which suppressed the expression levels of alphaSMA (*ACTA2*), connective tissue growth factor (*CTGF*), collagen type I (*COL1A1*), and type III collagen (*COL3A1*) in NRK49F cells [90].

*Liver fibrosis. Egr1* has been shown to contribute to liver fibrosis progression downstream of Elk-3 in CCl_4_-induced mouse liver fibrotic tissues and human liver cirrhotic tissues [148]. However, the beneficial effect of *Egr1* deletion in the context of liver fibrosis is contradictory. In an acute acetaminophen-induced liver injury mouse model, the inhibition on ERK1/2-mediated *Egr1* transcriptional activity attenuates hepatotoxicity, suggesting that inhibiting *Egr1* is beneficial to protect against liver fibrosis observed in long-term application of acetaminophen [149]. By contrast, another study shows that livers of *Egr1*^−/−^ mutant mice exhibit a more severe fibrotic response compared to those of wild-type mice under acetaminophen overdose [150]. The *Egr1* function remains elusive in liver fibrosis.

*Heart fibrosis.* EGR1 has been shown to be involved in cardiovascular homeostasis and diseases. Notably, *Egr1* transcription is activated in hypoxic and ischemic conditions in heart and in calcified heart valves [151,152]. The miR-150-5p retards the progression of myocardial fibrosis by targeting EGR1 [153].

As EGR1 is systematically mentioned as being involved in fibrosis progession in organ fibrosis animal models, *Egr1* is seen as a putative target to fight fibrosis. A recent antifibrotic chemical component has been identified with the PPARγ agonist (pioglitazone) that inhibits TGF-β-driven fibrosis in animal models for pulmonary, renal, and cardiac fibrosis, reviewed in [154]. Interestingly, pioglitazone has been shown to repress *Egr1* transcription and traduction in kidneys of TGF-β-driven renal fibrosis in mice [155] and in pancreas of a cerulein-induced acute pancreatitis mouse model [156].

### 4.5. EGR1 and Matrix Production in Rheumatoid Arthritis and Osteoarthritis 

Consistent with *Egr1* expression in cartilage and bone, the *Egr1* gene is reiteratively cited to be involved in chronic diseases that lead to articular cartilage degeneration, such as osteoarthritis and rheumatoid arthritis [157]. The molecular cascade underlying the pathogenesis of these two joint diseases are not well understood. Osteoarthritis leads to cartilage degeneration, reviewed in [158], while rheumatoid arthritis is an autoimmune and inflamatory disease associated with an increase of synovial fibroblasts leading to joint degeneration [159]. TNFα levels are increased in the synovial fluid of patients with osteoarthritis and rheumatoid arthritis [25] and reduce the expression of *Col2a1*, *Acan* (Aggrecan), and *Hapln1* through EGR1 recruitment to their promoters [25]. Classical and global transcriptomic analysis identified high *EGR1* expression in articular cartilage of patients with osteoarthritis [96,160,161] and in synovial tissues of rheumatoid arthritis patients [162,163,164]. Chondrocytes stimulation with interleukin-1β (IL-1) leads to the recruitment of EGR1 to *Pparg* promoter and downregulates its expression, preventing the protective effect of PPARγ in osteoarthritis [96]. Ectopic expression of EGR1 in articular cartilage aggravated the degradation of the cartilage matrix in mice [78]. The excess of *EGR1* induced an increase of transcripts and protein of type I collagen in synovial fibroblasts from rheumatoid arthritis patients [165]. *Egr1* represents a potential target for drug intervention in osteoarthritis or rheumatoid arthritis.

### 4.6. EGR1 and Scarred Tendon 

Abnormal tendon healing is frequent following tendon injury reviewed in [166,167]. Following accute rupture, tendons undergo a healing process involving the sequential and overlapping phases of inflamation, cell migration, cell proliferation, ECM production, and remodelling. These successive phases ultimately result in the production and spatial organization of type I collagen. However, the healing process is often incomplete in tendons, which leads to scar tendons that do not regain the mechanical properties of native tendons. The cellular basis of tendon fibrosis is not well understood and involves the contribution of intrinsic (tendon sheeths) and extrinsic (circulating cells) cell populations, recently rewiewed by [168]. The molecular basis underlying tendon fibrosis involves the main fibrotic signalling pathway, TGFβ [102], the transmenbrane protein TNMD [169], and the SCX transcription factor [170], which are also the main actors involved in tendon development [9,171,172]. Interestingly, SCX directly regulates the transcription of the *Acta2* gene (a fibrotic marker) in cardiac fibrosis [173]. Although *Egr1* is required for the correct transcriptional response in healing tendons [76], EGR1 function in the fibrotic response in tendon has not been established. However, given the EGR1 involvement in the fibrotic response in organs, EGR1 is very likely to be involved in tendon scarring.

## 5. Concluding Remarks

In addition to being involved in matrix production in normal conditions and fibrotic processes, EGR1 transcription factor has been associated with numerous cancers and has been shown to act as a tumor suppressor or a tumor promoter depending on cancer types, for reviews see [174,175]. The reason for this paradoxal/antagonistic EGR1 function depending on cancer types is not clear. Interestingly, EGR1 expression is correlated with prostate cancer progression and promotes prostate cancer metastases [176], which are associated with a massive increase of matrix [177]. *Egr1* has been already targeted to prevent the progression of prostate cancer carcinoma [178]. Interestingly, there is no associated cancer in tendons. Giant cell tumor of the tendon sheath (GCTTS) very rarely impacts tendon proper [179]. One attractive hypothesis is that the tendon matrix environement regulated by EGR1 is protective against cancer. 

In summary, the EGR1 transcription factor is a key checkpoint in the transcriptional response to external stimuli. Despite multiple regulatory elements in the *Egr1* promoter, *Egr1* has been repeatedly associated with matrix production in connective tissues in homeostatic and pathological conditions. *Egr1* deletion is a good therapeutic option for reducing fibrosis in many tissues. One attractive hypothesis is that EGR1 has a generic function in the transcription of matrix genes. Based on a recent report of EGR1 function in the brain, in the epigenetic control of the methylome during development and upon neuronal activity [180], EGR1 could act on the methylome of matrix genes in tendons and other connective tissues.

## Figures and Tables

**Table 1:** List of genes regulated by EGR1 in three connective tissues: tendon, bone, and adipose tissue.

Genes Regulated by EGR1 *Gene Names* (Protein Names)	Activated (+) or Repressed (−) by EGR1	References	Expression in Connective Tissues o	Physiological /Pathological Roles in Connective Tissues
**(I) Extracellular Proteins:** **(1) Extra Cellular Matrix (ECM) Components**				
*Acan* (Aggrecan)	**−**	[25]	Cartilage Tendon	ECM component of cartilage negatively regulated by EGR1
*Bgn* (Biglycan)	**+**	[46,76,84]	Tendon	ECM component of tendon Collagen fibrillogenesis
*Col1a1* * (1 chain of type I collagen)	**+**	[73,76,77,84,85,86,87,88,89,90]	Tendon Bone White adipose tissue	Main ECM structural and functional component of tendons ECM component of adipose tissue Fibrosis
*Col1a2* * (2 chain of type I collagen)	**+**	[46,76,77,85,91]	Tendon Bone White adipose tissue	Main ECM structural and functional component of tendons ECM component of adipose tissue Fibrosis
*Col2a1* * (1 chain of type II collagen)	**−**	[25,92]	Cartilage	Major ECM component of cartilage negatively regulated by EGR1
*Col3a1* (1 chain of type III collagen)	**+**	[73,76,77,86,87,88,90]	Tendon White adipose tissue	ECM component of tendon and adipose tissue Collagen fibrillogenesis Fibrosis
*Col5a1* (1 chain of type V collagen)	**+**	[73,76,77]	Tendon White adipose tissue	ECM component of tendon and adipose tissue Collagen fibrillogenesis
*Col5a2* (chain of type V collagen)	**+**	[77]	Tendon White adipose tissue	ECM component of tendon and adipose tissue Collagen fibrillogenesis
*Col6a1* (1 chain of type VI collagen)	**+**	[76]	Tendon White adipose tissue	ECM component of tendon and adipose tissue Fibrosis
*Col14a1* (1 chain of type XIV collagen)	**+**	[73,76,77]	Tendon White adipose tissue	ECM component of tendon and adipose tissue Collagen fibrillogenesis
*Dcn* (Decorin)	**+**	[76,77,84]	Tendon White adipose tissue	ECM component of tendon and adipose tissue Collagen fibrillogenesis
*Fbn1* (Fibrillin 1)	**+**	[76,77]	Tendon White adipose tissue	ECM component of tendon and adipose tissue Collagen fibrillogenesis
*Fn1* (Fibronectin)	**+**	[77]	Tendon White adipose tissue	ECM component of tendon and adipose tissue Matrix organizer Collagen fibrillogenesis
*Hapln1* * (Hyaluronan and proteoglycan link protein 1)	**−**	[25]	Cartilage Tendon	ECM component of cartilage negatively regulated by EGR1
*Postn* (Periostin)	**+**	[77]	Tendon Bone White adipose tissue	ECM component of tendon, bone, and adipose tissue
*Tnc* (Tenascin)	**+**	[76]	Tendon White adipose tissue	ECM component of tendon and adipose tissue Collagen fibrillogenesis
**(2) Secreted Proteins/Hormones**				
*Bglap* (Osteocalcin)	**+**	[93]	Bone	Bone-derived hormone involved in bone mineralization Regulation of glucose homeostasis
*Csf1* * (Macrophage Colony Stimulating Factor, M-CSF)	**−**	[80,81]	Bone Osteoclast precursors	Osteoclast proliferation and differentiation
*Ctsk* (Cathepsin K)	**+**	[77,93]	Bone Tendon Adipose tissue	ECM remodelling enzyme involved in bone formation during skeletal repair
*Lep* * (Leptin)	**+**	[41,77]	White adipose tissue	Hormone secreted by adipocytes involved in energy balance regulation
*Tgfb2* * (Transforming Growth Factor beta2, TGFbeta2)	**+**	[73,76,85]	Tendon	Tendon development, homeostasis, and repair fibrosis
**(II) Transmembrane Proteins**				
*Tnmd* (Tenomodulin)	**+**	[73,76,84,85]	Tendon White adipose tissue	Main tendon differentiation marker Transmembrane glycoprotein involved in tendon formation, homeostasis, and repair Increased in obese patients
**(III) Cytoplasmic Proteins**				
*Pnpla2* * (Adipose Triglyceride Lipase, ATGL)	**−**	[94]	White adipose tissue	Enzyme involved in adipose triglyceride lipolysis to mobilize triglyceride for energy production
*Acp5* (Tartrate-resistant acid phosphatase)	**+**	[93]	Bone	Metalloprotein enzyme involved in endochondral bone formation and repair
*Ucp1* * (Uncoupling protein 1)	**−**	[77]	White and brown adipose tissues	Thermogenic protein expressed in brown adipose tissue Upregulated in the context of white adipose tissue browning
**(IV) Transcription Factors/Nuclear Proteins**				
*Cebpb* * (CCAAT/Enhancer Binding Protein ß, C/EBPß)	**−**	[77]	White adipose tissue	Adipocyte differentiation Overexpression induces osteopenia
*Egr1* * (Early Growth Response 1)	**−**	[37]	Tendon Bone (hypertrophic cartilage) White adipose tissue	Zinc finger transcription factor involved in tendon formation, homeostasis, and repair *Egr1* deletion induces bone loss *Egr1* deletion induces white fat browning Fibrotic factor
*Foxc2* (Forkhead box protein C2)	**−**	[95]	Adipose tissue	Increases insulin sensitivity and is down-egulated in type 2 diabetic patients
*Mkx* (Mohawk)	**+**	[46]	Tendon	Homeobox protein involved in tendon formation and homeostasis
*Pparg* * (Peroxysome Proliferator Activated Receptor PPAR)	**−**	[96]	Adipose tissue Chondrocytes	Positive regulator of adipocyte differentiation Negative regulator of osteocyte differentiation Anti-fibrotic factor
*Scx* (Scleraxis)	**+**	[73,76,84,85]	Tendon	Main tendon marker bHLH transcription factor involved in tendon development, homeostasis, and repair

Genes marked with * are directly regulated by EGR1.

## References

[1] Santos A., Lagares D. (2018). Matrix Stiffness: The Conductor of Organ Fibrosis. Curr. Rheumatol. Rep..

[2] Nassari S., Duprez D., Fournier-Thibault C. (2017). Non-myogenic Contribution to Muscle Development and Homeostasis: The Role of Connective Tissues. Front. Cell Dev. Biol..

[3] Hannan R.T., Peirce S.M., Barker T.H. (2018). Fibroblasts: Diverse Cells Critical to Biomaterials Integration. ACS Biomater. Sci. Eng..

[4] Bi Y., Ehirchiou D., Kilts T.M., Inkson C.A., Embree M.C., Sonoyama W., Li L., Leet A.I., Seo B.-M., Zhang L. (2007). Identification of tendon stem/progenitor cells and the role of the extracellular matrix in their niche. Nat. Med..

[5] Mienaltowski M.J., Birk D.E. (2014). Structure, Physiology, and Biochemistry of Collagens. Adv. Exp. Med. Biol..

[6] Kadler K.E., Holmes D.F., Trotter J.A., Chapman J.A. (1996). Collagen fibril formation. Biochem. J..

[7] Huang A.H., Lu H.H., Schweitzer R. (2015). Molecular regulation of tendon cell fate during development. J. Orthop. Res..

[8] Dex S., Lin D., Shukunami C., Docheva D. (2016). Tenogenic modulating insider factor: Systematic assessment on the functions of tenomodulin gene. Gene.

[9] Murchison N.D., Price B.A., Conner D.A., Keene D.R., Olson E.N., Tabin C.J., Schweitzer R. (2007). Regulation of tendon differentiation by scleraxis distinguishes force-transmitting tendons from muscle-anchoring tendons. Development.

[10] Shukunami C., Takimoto A., Nishizaki Y., Yoshimoto Y., Tanaka S., Miura S., Watanabe H., Sakuma T., Yamamoto T., Kondoh G. (2018). Scleraxis is a transcriptional activator that regulates the expression of Tenomodulin, a marker of mature tenocytes and ligamentocytes. Sci. Rep..

[11] Levay A.K., Peacock J.D., Lu Y., Koch M., Hinton R.B., Kadler K.E., Lincoln J. (2008). Scleraxis is required for cell lineage differentiation and extracellular matrix remodeling during murine heart valve formation in vivo. Circ. Res..

[12] Mendias C.L., Gumucio J.P., Davis M.E., Bromley C.W., Davis C.S., Brooks S.V. (2012). Transforming growth factor-beta induces skeletal muscle atrophy and fibrosis through the induction of atrogin-1 and scleraxis. Muscle Nerve.

[13] Pryce B.A., Brent A.E., Murchison N.D., Tabin C.J., Schweitzer R. (2007). Generation of transgenic tendon reporters, ScxGFP and ScxAP, using regulatory elements of the scleraxis gene. Dev. Dyn. Off. Publ. Am. Assoc. Anat..

[14] Gaut L., Duprez D. (2016). Tendon development and diseases. Dev. Biol..

[15] Milet C., Duprez D. (2015). The Mkx homeoprotein promotes tenogenesis in stem cells and improves tendon repair. Ann. Transl. Med..

[16] Milbrandt J. (1987). A nerve growth factor-induced gene encodes a possible transcriptional regulatory factor. Science.

[17] Sukhatme V.P., Kartha S., Toback F.G., Taub R., Hoover R.G., Tsai-Morris C.H. (1987). A novel early growth response gene rapidly induced by fibroblast, epithelial cell and lymphocyte mitogens. Oncogene Res..

[18] Sukhatme V.P., Cao X., Chang L.C., Tsai-Morris C.H., Stamenkovich D., Ferreira P.C.P., Cohen D.R., Edwards S.A., Shows T.B., Curran T. (1988). A zinc finger-encoding gene coregulated with c-fos during growth and differentiation, and after cellular depolarization. Cell.

[19] Lim R.W., Varnum B.C., Herschman H.R. (1987). Cloning of tetradecanoyl phorbol ester-induced “primary response” sequences and their expression in density-arrested Swiss 3T3 cells and a TPA non-proliferative variant. Oncogene.

[20] Lemaire P., Relevant O., Bravo R., Charnay P. (1988). Two mouse genes encoding potential transcription factors with identical DNA-binding domains are activated by growth factors in cultured cells. Proc. Natl. Acad. Sci. USA.

[21] Christy B.A., Lau L.F., Nathans D. (1988). A gene activated in mouse 3T3 cells by serum growth factors encodes a protein with “zinc finger” sequences. Proc. Natl. Acad. Sci. USA.

[22] Mello C.V., Vicario D.S., Clayton D.F. (1992). Song presentation induces gene expression in the songbird forebrain. Proc. Natl. Acad. Sci. USA.

[23] Tsai-Morris C.H., Cao X.M., Sukhatme V.P. (1988). 5′ flanking sequence and genomic structure of Egr-1, a murine mitogen inducible zinc finger encoding gene. Nucleic Acids Res..

[24] Cao X., Guy G.R., Sukhatme V.P., Tan Y.H. (1992). Regulation of the Egr-1 gene by tumor necrosis factor and interferons in primary human fibroblasts. J. Biol. Chem..

[25] Rockel J.S., Bernier S.M., Leask A. (2009). Egr-1 inhibits the expression of extracellular matrix genes in chondrocytes by TNFα-induced MEK/ERK signalling. Arthritis Res. Ther..

[26] Granet C., Miossec P. (2004). Combination of the pro-inflammatory cytokines IL-1, TNF-α and IL-17 leads to enhanced expression and additional recruitment of AP-1 family members, Egr-1 and NF-κB in osteoblast-like cells. Cytokine.

[27] Geng L., Liao B., Jin L., Huang Z., Triggle C.R., Ding H., Zhang J., Huang Y., Lin Z., Xu A. (2019). Exercise Alleviates Obesity-Induced Metabolic Dysfunction via Enhancing FGF21 Sensitivity in Adipose Tissues. Cell Rep..

[28] Yang Y., Choi H., Seon M., Cho D., Bang S.I. (2016). LL-37 stimulates the functions of adipose-derived stromal/stem cells via early growth response 1 and the MAPK pathway. Stem Cell Res. Ther..

[29] Dalton S., Treisman R. (1992). Characterization of SAP-1, a protein recruited by serum response factor to the c-fos serum response element. Cell.

[30] Christy B., Nathans D. (1989). Functional serum response elements upstream of the growth factor-inducible gene zif268. Mol. Cell. Biol..

[31] McMahon S.B., Monroe J.G. (1995). A ternary complex factor-dependent mechanism mediates induction of egr-1 through selective serum response elements following antigen receptor cross-linking in B lymphocytes. Mol. Cell. Biol..

[32] Bhattacharyya S., Chen S.J., Wu M., Warner-Blankenship M., Ning H., Lakos G., Mori Y., Chang E., Nihijima C., Takehara K. (2008). Smad-independent transforming growth factor-β regulation of early growth response-1 and sustained expression in fibrosis: Implications for scleroderma. Am. J. Pathol..

[33] Hoffmann E., Ashouri J., Wolter S., Doerrie A., Dittrich-Breiholz O., Schneider H., Wagner E.F., Troppmair J., Mackman N., Kracht M. (2008). Transcriptional regulation of EGR-1 by the interleukin-1-JNK-MKK7-c-Jun pathway. J. Biol. Chem..

[34] Hess J., Angel P., Schorpp-Kistner M. (2004). AP-1 subunits: Quarrel and harmony among siblings. J. Cell Sci..

[35] Aicher W.K., Sakamoto K.M., Hack A., Eibel H. (1999). Analysis of functional elements in the human Egr-1 gene promoter. Rheumatol. Int..

[36] Cao X.M., Koski R.A., Gashler A., McKiernan M., Morris C.F., Gaffney R., Hay R.V., Sukhatme V.P. (1990). Identification and characterization of the Egr-1 gene product, a DNA-binding zinc finger protein induced by differentiation and growth signals. Mol. Cell. Biol..

[37] Cao X., Mahendran R., Guy G.R., Tan Y.H. (1993). Detection and characterization of cellular EGR-1 binding to its recognition site. J. Biol. Chem..

[38] Sheng M., McFadden G., Greenberg M.E. (1990). Membrane depolarization and calcium induce c-fos transcription via phosphorylation of transcription factor CREB. Neuron.

[39] Vaccarino F.M., Hayward M.D., Le H.N., Hartigan D.J., Duman R.S., Nestler E.J. (1993). Induction of immediate early genes by cyclic AMP in primary cultures of neurons from rat cerebral cortex. Mol. Brain Res..

[40] Kang J.H., Kim M.J., Jang H.I., Koh K.H., Yum K.S., Rhie D.J., Shin H.Y., Sang J.H., Kim M.S., Jo Y.H. (2007). Proximal cyclic AMP response element is essential for exendin-4 induction of rat EGR-1 gene. Am. J. Physiol. Endocrinol. Metab..

[41] Mohtar O., Ozdemir C., Roy D., Shantaram D., Emili A., Kandror K.V. (2019). Egr1 mediates the effect of insulin on leptin transcription in adipocytes. J. Biol. Chem..

[42] Saltiel A.R., Kahn C.R. (2001). Insulin signalling and the regulation of glucose and lipid metabolism. Nature.

[43] Biddinger S.B., Kahn C.R. (2006). FROM MICE TO MEN: Insights into the Insulin Resistance Syndromes. Annu. Rev. Physiol..

[44] Franke T.F., Yang S.I., Chan T.O., Datta K., Kazlauskas A., Morrison D.K., Kaplan D.R., Tsichlis P.N. (1995). The protein kinase encoded by the Akt proto-oncogene is a target of the PDGF-activated phosphatidylinositol 3-kinase. Cell.

[45] Müller I., Lipp P., Thiel G. (2012). Ca2+ signaling and gene transcription in glucose-stimulated insulinoma cells. Cell Calcium.

[46] Wu Y.F., Wang H.K., Chang H.W., Sun J., Sun J.S., Chao Y.H. (2017). High glucose alters tendon homeostasis through downregulation of the AMPK/Egr1 pathway. Sci. Rep..

[47] Huang R.P., Yan F., Boynton A.L. (1999). UV irradiation upregulates Egr-1 expression at transcription level. J. Cell. Biochem..

[48] Thyss R., Virolle V., Imbert V., Peyron J.F., Aberdam D., Virolle T. (2005). NF-κB/Egr-1/Gadd45 are sequentially activated upon UVB irradiation to mediate epidermal cell death. EMBO J..

[49] Trinh N.T., Yamashita T., Ohneda K., Kimura K., Salazar G.T.A., Sato F., Ohneda O. (2016). Increased Expression of EGR-1 in Diabetic Human Adipose Tissue-Derived Mesenchymal Stem Cells Reduces Their Wound Healing Capacity. Stem Cells Dev..

[50] Schwachtgen J.L., Houston P., Campbell C., Sukhatme V., Braddock M. (1998). Fluid shear stress activation of egr-1 transcription in cultured human endothelial and epithelial cells is mediated via the extracellular signal-related kinase 1/2 mitogen-activated protein kinase pathway. J. Clin. Invest..

[51] Yu J., Zhang S.S., Saito K., Williams S., Arimura Y., Ma Y., Ke Y., Baron V., Mercola D., Feng G.S. (2009). PTEN regulation by Akt-EGR1-ARF-PTEN axis. EMBO J..

[52] Christy B., Nathans D. (1989). DNA binding site of the growth factor-inducible protein Zif268. Proc. Natl. Acad. Sci. USA.

[53] Lemaire P., Vesque C., Schmitt J., Stunnenberg H., Frank R., Charnay P. (1990). The serum-inducible mouse gene Krox-24 encodes a sequence-specific transcriptional activator. Mol. Cell. Biol..

[54] Gashler A.L., Swaminathan S., Sukhatme V.P. (1993). A novel repression module, an extensive activation domain, and a bipartite nuclear localization signal defined in the immediate-early transcription factor Egr-1. Mol. Cell. Biol..

[55] Russo M.W., Sevetson B.R., Milbrandt J. (1995). Identification of NAB1, a repressor of NGFI-A- and Krox20-mediated transcription. Proc. Natl. Acad. Sci. USA.

[56] Svaren J., Sevetson B.R., Apel E.D., Zimonjic D.B., Popescu N.C., Milbrandt J. (1996). NAB2, a corepressor of NGFI-A (Egr-1) and Krox20, is induced by proliferative and differentiative stimuli. Mol. Cell. Biol..

[57] Sevetson B.R., Svaren J., Milbrandt J. (2000). A novel activation function for NAB proteins in EGR-dependent transcription of the luteinizing hormone β gene. J. Biol. Chem..

[58] Cao X., Mahendran R., Guy G.R., Tan Y.H. (1992). Protein phosphatase inhibitors induce the sustained expression of the Egr- 1 gene and the hyperphosphorylation of its gene product. J. Biol. Chem..

[59] Huang R.P., Fan Y., DeBelle I., Ni Z., Matheny W., Adamson E.D. (1998). Egr-1 inhibits apoptosis during the UV response: Correlation of cell survival with Egr-1 phosphorylation. Cell Death Differ..

[60] Huang R.P., Fan Y., Peng A., Zeng Z.L., Reed J.C., Adamson E.D., Boynton A.L. (1998). Suppression of human fibrosarcoma cell growth by transcription factor, Egr-1, involves down-regulation of Bcl-2. Int. J. Cancer.

[61] Jain N., Mahendran R., Philp R., Guy G.R., Tan Y.H., Cao X. (1996). Casein kinase II associates with Egr-1 and acts as a negative modulator of its DNA binding and transcription activities in NIH 3T3 cells. J. Biol. Chem..

[62] Yu J., De Belle I., Liang H., Adamson E.D. (2004). Coactivating factors p300 and CBP are transcriptionally crossregulated by Egr1 in prostate cells, leading to divergent responses. Mol. Cell.

[63] Bae M.-H., Jeong C.-H., Kim S.-H., Bae M.-K., Jeong J.-W., Ahn M.-Y., Bae S.-K., Kim N.D., Kim C.W., Kim K.-R. (2002). Regulation of Egr-1 by association with the proteasome component C8. Biochim. Biophys. Acta.

[64] Manente A.G., Pinton G., Tavian D., Lopez-Rodas G., Brunelli E., Moro L. (2011). Coordinated sumoylation and ubiquitination modulate EGF induced EGR1 expression and stability. PLoS ONE.

[65] Bai X.C., Lu D., Bai J., Zheng H., Ke Z.Y., Li X.M., Luo S.Q. (2004). Oxidative stress inhibits osteoblastic differentiation of bone cells by ERK and NF-κB. Biochem. Biophys. Res. Commun..

[66] Garrett I.R., Boyce B.F., Oreffo R.O.C., Bonewald L., Poser J., Mundy G.R. (1990). Oxygen-derived free radicals stimulate osteoclastic bone resorption in rodent bone in vitro and in vivo. J. Clin. Invest..

[67] Key L.L., Wolf W.C., Gundberg C.M., Ries W.L. (1994). Superoxide and bone resorption. Bone.

[68] Mody N., Parhami F., Sarafian T.A., Demer L.L. (2001). Oxidative stress modulates osteoblastic differentiation of vascular and bone cells. Free Radic. Biol. Med..

[69] Nose K., Shibanuma M., Kikuchi K., Kageyama H., Sakiyama S., Kuroki T. (1991). Transcriptional activation of early-response genes by hydrogen peroxide in a mouse osteoblastic cell line. Eur. J. Biochem..

[70] Pines A., Bivi N., Romanello M., Damante G., Kelley M.R., Adamson E.D., D’Andrea P., Quadrifoglio F., Moro L., Tell G. (2005). Cross-regulation between Egr-1 and APE/Ref-1 during early response to oxidative stress in the human osteoblastic HOBIT cell line: Evidence for an autoregulatory loop. Free Radic. Res..

[71] Huang R.P., Adamson E.D. (1993). Characterization of the DNA-Binding Properties of the Early Growth Response-1 (Egr-1) Transcription Factor: Evidence for Modulation by a Redox Mechanism. DNA Cell Biol..

[72] McMahon A.P., Champion J.E., McMahon J.A., Sukhatme V.P. (1990). Developmental expression of the putative transcription factor Egr-1 suggests that Egr-1 and c-fos are coregulated in some tissues. Development.

[73] Lejard V., Blais F., Guerquin M.-J., Bonnet A., Bonnin M.-A., Havis E., Malbouyres M., Bidaud C.B., Maro G., Gilardi-Hebenstreit P. (2011). EGR1 and EGR2 involvement in vertebrate tendon differentiation. J. Biol. Chem..

[74] Orgeur M., Martens M., Leonte G., Nassari S., Bonnin M.A., Börno S.T., Timmermann B., Hecht J., Duprez D., Stricker S. (2018). Genome-wide strategies identify downstream target genes of chick connective tissue-associated transcription factors. Development.

[75] Yue F., Cheng Y., Breschi A., Vierstra J., Wu W., Ryba T., Sandstrom R., Ma Z., Davis C., Pope B.D. (2014). A comparative encyclopedia of DNA elements in the mouse genome. Nature.

[76] Guerquin M.-J., Charvet B., Nourissat G., Havis E., Ronsin O., Bonnin M.-A., Ruggiu M., Olivera-Martinez I., Robert N., Lu Y. (2013). Transcription factor EGR1 directs tendon differentiation and promotes tendon repair. J. Clin. Invest..

[77] Milet C., Bléher M., Allbright K., Orgeur M., Coulpier F., Duprez D., Havis E. (2017). Egr1 deficiency induces browning of inguinal subcutaneous white adipose tissue in mice. Sci. Rep..

[78] Sun X., Huang H., Pan X., Li S., Xie Z., Ma Y., Hu B., Wang J., Chen Z., Shi P. (2019). EGR1 promotes the cartilage degeneration and hypertrophy by activating the Krüppel-like factor 5 and β-catenin signaling. Biochim. Biophys. Acta. Mol. basis Dis..

[79] Chen Z., Yue S.X., Zhou G., Greenfield E.M., Murakami S. (2015). ERK1 and ERK2 regulate chondrocyte terminal differentiation during endochondral bone formation. J. Bone Miner. Res..

[80] Srivastava S., Weitzmann M.N., Kimble R.B., Rizzo M., Zahner M., Milbrandt J., Ross F.P., Pacifici R. (1998). Estrogen blocks M-CSF gene expression and osteoclast formation by regulating phosphorylation of Egr-1 and its interaction with Sp-1. J. Clin. Invest..

[81] Cenci S., Weitzmann M.N., Gentile M.A., Aisa M.C., Pacifici R. (2000). M-CSF neutralization and Egr-1 deficiency prevent ovariectomy-induced bone loss. J. Clin. Invest..

[82] Lee S.L., Tourtellotte L.C., Wesselschmidt R.L., Milbrandt J. (1995). Growth and differentiation proceeds normally in cells deficient in the immediate early gene NGFI-A. J. Biol. Chem..

[83] Topilko P., Schneider-Maunoury S., Levi G., Trembleau A., Gourdji D., Driancourt M.A., Rao C.V., Charnay P. (1998). Multiple pituitary and ovarian defects in Krox-24 (NGFI-A, Egr-1)-targeted mice. Mol. Endocrinol..

[84] Han W., Wang B., Liu J., Chen L. (2017). The p16/miR-217/EGR1 pathway modulates age-related tenogenic differentiation in tendon stem/progenitor cells. Acta Biochim. Biophys. Sin. (Shanghai).

[85] Gaut L., Robert N., Delalande A., Bonnin M.A., Pichon C., Duprez D. (2016). EGR1 regulates transcription downstream of mechanical signals during tendon formation and healing. PLoS ONE.

[86] Halberg N., Khan T., Trujillo M.E., Wernstedt-Asterholm I., Attie A.D., Sherwani S., Wang Z.V., Landskroner-Eiger S., Dineen S., Magalang U.J. (2009). Hypoxia-Inducible Factor 1 Induces Fibrosis and Insulin Resistance in White Adipose Tissue. Mol. Cell. Biol..

[87] Sun K., Halberg N., Khan M., Magalang U.J., Scherer P.E. (2013). Selective Inhibition of Hypoxia-Inducible Factor 1 Ameliorates Adipose Tissue Dysfunction. Mol. Cell. Biol..

[88] Ackerman J.E., Geary M.B., Orner C.A., Bawany F., Loiselle A.E. (2017). Obesity/Type II diabetes alters macrophage polarization resulting in a fibrotic tendon healing response. PLoS ONE.

[89] Wu M., Melichian D.S., De La Garza M., Gruner K., Bhattacharyya S., Barr L., Nair A., Shahrara S., Sporn P.H.S., Mustoe T.A. (2009). Essential roles for early growth response transcription factor Egr-1 in tissue fibrosis and wound healing. Am. J. Pathol..

[90] Zhang X., Yang Z., Heng Y., Miao C. (2019). MicroRNA-181 exerts an inhibitory role during renal fibrosis by targeting early growth response factor-1 and attenuating the expression of profibrotic markers. Mol. Med. Rep..

[91] Chen S.J., Ning H., Ishida W., Sodin-Semrl S., Takagawa S., Mori Y., Varga J. (2006). The early-immediate gene EGR-1 is induced by transforming growth factor-β and mediates stimulation of collagen gene expression. J. Biol. Chem..

[92] Tan L., Peng H., Osaki M., Choy B.K., Auron P.E., Sandell L.J., Goldring M.B. (2003). Egr-1 mediates transcriptional repression of COL2A1 promoter activity by interleukin-1β. J. Biol. Chem..

[93] Reumann M.K., Strachna O., Yagerman S., Torrecilla D., Kim J., Doty S.B., Lukashova L., Boskey A.L., Mayer-Kuckuk P. (2011). Loss of transcription factor early growth response gene 1 results in impaired endochondral bone repair. Bone.

[94] Chakrabarti P., Kim J.Y., Singh M., Shin Y.-K., Kim J., Kumbrink J., Wu Y., Lee M.-J., Kirsch K.H., Fried S.K. (2013). Insulin inhibits lipolysis in adipocytes via the evolutionarily conserved mTORC1-Egr1-ATGL-mediated pathway. Mol. Cell. Biol..

[95] Zhang J., Zhang Y., Sun T., Guo F., Huang S., Chandalia M., Abate N., Fan D., Xin H.-B., Chen Y.E. (2013). Dietary obesity-induced Egr-1 in adipocytes facilitates energy storage via suppression of FOXC2. Sci. Rep..

[96] Nebbaki S.-S., El Mansouri F.E., Afif H., Kapoor M., Benderdour M., Duval N., Pelletier J.-P., Martel-Pelletier J., Fahmi H. (2012). Egr-1 contributes to IL-1-mediated down-regulation of peroxisome proliferator-activated receptor γ expression in human osteoarthritic chondrocytes. Arthritis Res. Ther..

[97] Tao X., Liu J., Chen L., Zhou Y., Tang K. (2015). EGR1 Induces Tenogenic Differentiation of Tendon Stem Cells and Promotes Rabbit Rotator Cuff Repair. Cell. Physiol. Biochem..

[98] Wang B., Guo J., Feng L., Suen C.-W., Fu W.-M., Zhang J.-F., Li G. (2016). MiR124 suppresses collagen formation of human tendon derived stem cells through targeting egr1. Exp. Cell Res..

[99] Qiu S., Sun Y., Xu J., Wen G., Yu Y., Wu T., Chai Y. (2019). Ferulic acid improves self-renewal and differentiation of human tendon-derived stem cells by upregulating early growth response 1 through hypoxia. Genesis.

[100] Eliasson P., Andersson T., Hammerman M., Aspenberg P. (2013). Primary gene response to mechanical loading in healing rat Achilles tendons. J. Appl. Physiol..

[101] Eliasson P., Andersson T., Aspenberg P. (2009). Rat Achilles tendon healing: Mechanical loading and gene expression. J. Appl. Physiol..

[102] Maeda T., Sakabe T., Sunaga A., Sakai K., Rivera A.L., Keene D.R., Sasaki T., Stavnezer E., Iannotti J., Schweitzer R. (2011). Conversion of mechanical force into TGF-β-mediated biochemical signals. Curr. Biol..

[103] Derby B.M., Reichensperger J., Chambers C., Bueno R.A., Suchy H., Neumeister M.W. (2012). Early growth response factor-1: Expression in a rabbit flexor tendon scar model. Plast. Reconstr. Surg..

[104] Hammerman M., Aspenberg P., Eliasson P. (2014). Microtrauma stimulates rat Achilles tendon healing via an early gene expression pattern similar to mechanical loading. J. Appl. Physiol..

[105] Yeung C.-Y.C., Kadler K.E. (2019). Chapter Eleven - Importance of the circadian clock in tendon development. Curr. Top. Dev. Biol..

[106] Chang J., Garva R., Pickard A., Yeung C.-Y.C., Mallikarjun V., Swift J., Holmes D.F., Calverley B., Lu Y., Adamson A. (2020). Circadian control of the secretory pathway maintains collagen homeostasis. Nat. Cell Biol..

[107] Riedel C.S., Georg B., Jørgensen H.L., Hannibal J., Fahrenkrug J. (2018). Mice Lacking EGR1 Have Impaired Clock Gene (BMAL1) Oscillation, Locomotor Activity, and Body Temperature. J. Mol. Neurosci..

[108] Magnusson S.P., Kjaer M. (2019). The impact of loading, unloading, ageing and injury on the human tendon. J. Physiol..

[109] Schiele N.R., Marturano J.E., Kuo C.K. (2013). Mechanical factors in embryonic tendon development: Potential cues for stem cell tenogenesis. Curr. Opin. Biotechnol..

[110] Arvind V., Huang A.H. (2017). Mechanobiology of limb musculoskeletal development. Ann. N. Y. Acad. Sci..

[111] Marturano J.E., Arena J.D., Schiller Z.A., Georgakoudi I., Kuo C.K. (2013). Characterization of mechanical and biochemical properties of developing embryonic tendon. Proc. Natl. Acad. Sci. USA.

[112] Havis E., Bonnin M.-A., De Lima J.E., Charvet B., Milet C., Duprez D. (2016). TGFβ and FGF promote tendon progenitor fate and act downstream of muscle contraction to regulate tendon differentiation during chick limb development. Development.

[113] Herchenhan A., Dietrich-Zagonel F., Schjerling P., Kjær M., Eliasson P. (2019). Early Growth Response Genes Increases Rapidly After Mechanical Overloading and Unloading in Tendon Constructs. J. Orthop. Res..

[114] Yang F., Zhang A., Richardson D.W. (2019). Regulation of the tenogenic gene expression in equine tenocyte-derived induced pluripotent stem cells by mechanical loading and Mohawk. Stem Cell Res..

[115] Aspenberg P. (2007). Stimulation of tendon repair: Mechanical loading, GDFs and platelets. A mini-review. Int. Orthop..

[116] Gumucio J.P., Sugg K.B., Mendias C.L. (2015). TGF-β Superfamily Signaling in Muscle and Tendon Adaptation to Resistance Exercise. Exerc. Sport Sci. Rev..

[117] Weatherholt A.M., Fuchs R.K., Warden S.J. (2012). Specialized connective tissue: Bone, the structural framework of the upper extremity. J. Hand Ther..

[118] Reumann M.K., Strachna O., Lukashova L., Verdelis K., Donnelly E., Boskey A.L., Mayer-Kuckuk P. (2011). Early growth response gene 1 regulates bone properties in mice. Calcif. Tissue Int..

[119] Lu K., Shi T.-S., Shen S.-Y., Lu W.-L., Wu J., Zhang K.-J., Zhu X.-B., Shi Y., Liu X.-L., Yu F. (2018). Egr1 deficiency disrupts dynamic equilibrium of chondrocyte extracellular matrix through PPARγ/RUNX2 signaling pathways. Am. J. Transl. Res..

[120] Karsenty G., Wagner E.F. (2002). Reaching a genetic and molecular understanding of skeletal development. Dev. Cell.

[121] Marcelin G., Clément K. (2018). Adipose tissue fibrosis: An aggravating factor in obesity. Medecine/Sciences.

[122] Khan T., Muise E.S., Iyengar P., Wang Z.V., Chandalia M., Abate N., Zhang B.B., Bonaldo P., Chua S., Scherer P.E. (2009). Metabolic Dysregulation and Adipose Tissue Fibrosis: Role of Collagen VI. Mol. Cell. Biol..

[123] Ruiz-Ojeda F.J., Méndez-Gutiérrez A., Aguilera C.M., Plaza-Díaz J. (2019). Extracellular matrix remodeling of adipose tissue in obesity and metabolic diseases. Int. J. Mol. Sci..

[124] Wang Q.A., Tao C., Gupta R.K., Scherer P.E. (2013). Tracking adipogenesis during white adipose tissue development, expansion and regeneration. Nat. Med..

[125] Bartelt A., Heeren J. (2014). Adipose tissue browning and metabolic health. Nat. Rev. Endocrinol..

[126] Hinz B., Lagares D. (2020). Evasion of apoptosis by myofibroblasts: A hallmark of fibrotic diseases. Nat. Rev. Rheumatol..

[127] Vallée A., Lecarpentier Y. (2019). TGF-β in fibrosis by acting as a conductor for contractile properties of myofibroblasts. Cell Biosci..

[128] Buechler C., Krautbauer S., Eisinger K. (2015). Adipose tissue fibrosis. World J. Diabetes.

[129] Sun K., Tordjman J., Clément K., Scherer P.E. (2013). Fibrosis and adipose tissue dysfunction. Cell Metab..

[130] Sun K., Kusminski C.M., Scherer P.E., Sun K., Kusminski C.M., Scherer P.E. (2011). Adipose tissue remodeling and obesity Find the latest version: Review series Adipose tissue remodeling and obesity. J. Clin. Invest..

[131] Divoux A., Clement K. (2011). Architecture and the extracellular matrix: The still unappreciated components of the adipose tissue. Obes. Rev..

[132] Lackey D.E., Burk D.H., Ali M.R., Mostaedi R., Smith W.H., Park J., Scherer P.E., Seay S.A., McCoin C.S., Bonaldo P. (2014). Contributions of adipose tissue architectural and tensile properties toward defining healthy and unhealthy obesity. Am. J. Physiol. Endocrinol. Metab..

[133] Yu X., Shen N., Zhang M.-L., Pan F.-Y., Wang C., Jia W.-P., Liu C., Gao Q., Gao X., Xue B. (2011). Egr-1 decreases adipocyte insulin sensitivity by tilting PI3K/Akt and MAPK signal balance in mice. EMBO J..

[134] Saiki A., Olsson M., Jernås M., Gummesson A., McTernan P.G., Andersson J., Jacobson P., Sjöholm K., Olsson B., Yamamura S. (2009). Tenomodulin Is Highly Expressed in Adipose Tissue, Increased in Obesity, and Down-Regulated during Diet-Induced Weight Loss. J. Clin. Endocrinol. Metab..

[135] Aguilera C.M., Gomez-Llorente C., Tofe I., Gil-Campos M., Cañete R., Gil Á. (2015). Genome-wide expression in visceral adipose tissue from obese prepubertal children. Int. J. Mol. Sci..

[136] Senol-Cosar O., Flach R.J.R., DiStefano M., Chawla A., Nicoloro S., Straubhaar J., Hardy O.T., Noh H.L., Kim J.K., Wabitsch M. (2016). Tenomodulin promotes human adipocyte differentiation and beneficial visceral adipose tissue expansion. Nat. Commun..

[137] Ranger T.A., Wong A.M.Y., Cook J.L., Gaida J.E. (2016). Is there an association between tendinopathy and diabetes mellitus? A systematic review with meta-analysis. Br. J. Sports Med..

[138] Nichols A.E.C., Oh I., Loiselle A.E. (2019). Effects of Type II Diabetes Mellitus on Tendon Homeostasis and Healing. J. Orthop. Res..

[139] Lin Y.-C., Li Y.-J., Rui Y.-F., Dai G.-C., Shi L., Xu H.-L., Ni M., Zhao S., Chen H., Wang C. (2017). The effects of high glucose on tendon-derived stem cells: Implications of the pathogenesis of diabetic tendon disorders. Oncotarget.

[140] Van Caam A., Vonk M., van den Hoogen F., van Lent P., van der Kraan P. (2018). Unraveling SSc Pathophysiology; The Myofibroblast. Front. Immunol..

[141] Korman B. (2019). Evolving insights into the cellular and molecular pathogenesis of fibrosis in systemic sclerosis. Transl. Res..

[142] Bhattacharyya S., Wu M., Fang F., Tourtellotte W., Feghali-Bostwick C., Varga J. (2011). Early growth response transcription factors: Key mediators of fibrosis and novel targets for anti-fibrotic therapy. Matrix Biol..

[143] Bhattacharyya S., Fang F., Tourtellotte W., Varga J. (2013). Egr-1: New conductor for the tissue repair orchestra directs harmony (regeneration) or cacophony (fibrosis). J. Pathol..

[144] Bhattacharyya S., Sargent J.L., Du P., Lin S., Tourtellotte W.G., Takehara K., Whitfield M.L., Varga J. (2011). Egr-1 induces a profibrotic injury/repair gene program associated with systemic sclerosis. PLoS ONE.

[145] Yasuoka H., Hsu E., Ruiz X.D., Steinman R.A., Choi A.M.K., Feghali-Bostwick C.A. (2009). The fibrotic phenotype induced by IGFBP-5 is regulated by MAPK activation and egr-1-dependent and -independent mechanisms. Am. J. Pathol..

[146] Ghatak S., Markwald R.R., Hascall V.C., Dowling W., Lottes R.G., Baatz J.E., Beeson G., Beeson C.C., Perrella M.A., Thannickal V.J. (2017). Transforming growth factor β1 (TGF β1) regulates CD44V6 expression and activity through extracellular signal-regulated kinase (ERK)-induced EGR1 in pulmonary fibrogenic fibroblasts. J. Biol. Chem..

[147] Ho L.C., Sung J.M., Shen Y.T., Jheng H.F., Chen S.H., Tsai P.J., Tsai Y.S. (2016). Egr-1 deficiency protects from renal inflammation and fibrosis. J. Mol. Med..

[148] Li T.Z., Kim S.M., Hur W., Choi J.E., Kim J.-H., Hong S.W., Lee E.B., Lee J.H., Yoon S.K. (2017). Elk-3 Contributes to the Progression of Liver Fibrosis by Regulating the Epithelial-Mesenchymal Transition. Gut Liver.

[149] Peng W.-X., Xiong E.-M., Ge L., Wan Y.-Y., Zhang C.-L., Du F.-Y., Xu M., Bhat R.A., Jin J., Gong A.-H. (2016). Egr-1 promotes hypoxia-induced autophagy to enhance chemo-resistance of hepatocellular carcinoma cells. Exp. Cell Res..

[150] Bai Q., Yan H., Sheng Y., Jin Y., Shi L., Ji L., Wang Z. (2017). Long-term acetaminophen treatment induced liver fibrosis in mice and the involvement of Egr-1. Toxicology.

[151] Gess B., Wolf K., Pfeifer M., Riegger G.A., Kurtz A. (1997). In vivo carbon monoxide exposure and hypoxic hypoxia stimulate immediate early gene expression. Pflugers Arch..

[152] Ghazvini-Boroujerdi M., Clark J., Narula N., Palmatory E., Connolly J.M., DeFelice S., Xu J., Jian B., Hazelwood S., Levy R.J. (2004). Transcription factor Egr-1 in calcific aortic valve disease. J. Heart Valve Dis..

[153] Shen J., Xing W., Gong F., Wang W., Yan Y., Zhang Y., Xie C., Fu S. (2019). MiR-150-5p retards the progression of myocardial fibrosis by targeting EGR1. Cell Cycle.

[154] Kökény G., Calvier L., Legchenko E., Chouvarine P., Mózes M.M., Hansmann G. (2020). PPARγ is a gatekeeper for extracellular matrix and vascular cell homeostasis: Beneficial role in pulmonary hypertension and renal/cardiac/pulmonary fibrosis. Curr. Opin. Nephrol. Hypertens..

[155] Németh Á., Mózes M.M., Calvier L., Hansmann G., Kökény G. (2019). The PPARγ agonist pioglitazone prevents TGF-β induced renal fibrosis by repressing EGR-1 and STAT3. BMC Nephrol..

[156] Wan H., Yuan Y., Liu J., Chen G. (2012). Pioglitazone, a PPAR-γ activator, attenuates the severity of cerulein-induced acute pancreatitis by modulating early growth response-1 transcription factor. Transl. Res..

[157] Li G., Han N., Li Z., Lu Q. (2013). Identification of transcription regulatory relationships in rheumatoid arthritis and osteoarthritis. Clin. Rheumatol..

[158] Kloppenburg M., Berenbaum F. (2020). Osteoarthritis year in review 2019: Epidemiology and therapy. Osteoarthr. Cartil..

[159] Huber L.C., Distler O., Tarner I., Gay R.E., Gay S., Pap T. (2006). Synovial fibroblasts: Key players in rheumatoid arthritis. Rheumatology.

[160] Zhang X., Yuan Z., Cui S. (2016). Identifying candidate genes involved in osteoarthritis through bioinformatics analysis. Clin. Exp. Rheumatol..

[161] Feng Z., Lian K.-J. (2015). Identification of genes and pathways associated with osteoarthritis by bioinformatics analyses. Eur. Rev. Med. Pharmacol. Sci..

[162] Grimbacher B., Aicher W.K., Peter H.H., Eibel H. (1997). Measurement of transcription factor c-fos and EGR-1 mRNA transcription levels in synovial tissue by quantitative RT-PCR. Rheumatol. Int..

[163] Trabandt A., Aicher W.K., Gay R.E., Sukhatme V.P., Fassbender H.G., Gay S. (1992). Spontaneous expression of immediately-early response genes c-fos and egr-1 in collagenase-producing rheumatoid synovial fibroblasts. Rheumatol. Int..

[164] Aicher W.K., Heer A.H., Trabandt A., Bridges Jr S.L., Schroeder Jr H.W., Stransky G., Gay R.E., Eibel H., Peter H.H., Siebenlist U. (1994). Overexpression of zinc-finger transcription factor Z-225/Egr-1 in synoviocytes from rheumatoid arthritis patients. J. Immunol..

[165] Alexander D., Judex M., Meyringer H., Weis-Klemm M., Gay B., Müller-Ladner U., Aicher W.K. (2002). Transcription factor Egr-1 activates collagen expression in immortalized fibroblasts or fibrosarcoma cells. Biol. Chem..

[166] Nourissat G., Berenbaum F., Duprez D. (2015). Tendon injury: From biology to tendon repair. Nat. Rev. Rheumatol..

[167] Docheva D., Müller S.A., Majewski M., Evans C.H. (2015). Biologics for tendon repair. Adv. Drug Deliv. Rev..

[168] Nichols A.E.C., Best K.T., Loiselle A.E. (2019). The cellular basis of fibrotic tendon healing: Challenges and opportunities. Transl. Res..

[169] Lin D., Alberton P., Caceres M.D., Volkmer E., Schieker M., Docheva D. (2017). Tenomodulin is essential for prevention of adipocyte accumulation and fibrovascular scar formation during early tendon healing. Cell Death Dis..

[170] Sakabe T., Sakai K., Maeda T., Sunaga A., Furuta N., Schweitzer R., Sasaki T., Sakai T. (2018). Transcription factor scleraxis vitally contributes to progenitor lineage direction in wound healing of adult tendon in mice. J. Biol. Chem..

[171] Pryce B.A., Watson S.S., Murchison N.D., Staverosky J.A., Dünker N., Schweitzer R. (2009). Recruitment and maintenance of tendon progenitors by TGFbeta signaling are essential for tendon formation. Development.

[172] Docheva D., Hunziker E.B., Fässler R., Brandau O. (2005). Tenomodulin is necessary for tenocyte proliferation and tendon maturation. Mol. Cell. Biol..

[173] Bagchi R.A., Roche P., Aroutiounova N., Espira L., Abrenica B., Schweitzer R., Czubryt M.P. (2016). The transcription factor scleraxis is a critical regulator of cardiac fibroblast phenotype. BMC Biol..

[174] Pagel J.-I., Deindl E. (2011). Early growth response 1—a transcription factor in the crossfire of signal transduction cascades. Indian J. Biochem. Biophys..

[175] Magee N., Zhang Y. (2017). Role of early growth response 1 in liver metabolism and liver cancer. Hepatoma Res..

[176] Li L., Ameri A.H., Wang S., Jansson K.H., Casey O.M., Yang Q., Beshiri M.L., Fang L., Lake R.G., Agarwal S. (2019). EGR1 regulates angiogenic and osteoclastogenic factors in prostate cancer and promotes metastasis. Oncogene.

[177] Penet M.-F., Kakkad S., Pathak A.P., Krishnamachary B., Mironchik Y., Raman V., Solaiyappan M., Bhujwalla Z.M. (2017). Structure and Function of a Prostate Cancer Dissemination-Permissive Extracellular Matrix. Clin. Cancer Res..

[178] Gitenay D., Baron V.T. (2009). Is EGR1 a potential target for prostate cancer therapy?. Future Oncol..

[179] Zhao Q., Lu H. (2019). Giant cell tumor of tendon sheath in the wrist that damaged the extensor indicis proprius tendon: A case report and literature review. BMC Cancer.

[180] Sun Z., Xu X., He J., Murray A., Sun M.-A., Wei X., Wang X., McCoig E., Xie E., Jiang X. (2019). EGR1 recruits TET1 to shape the brain methylome during development and upon neuronal activity. Nat. Commun..

